# *Escherichia coli* RIC Is Able to Donate Iron to Iron-Sulfur Clusters

**DOI:** 10.1371/journal.pone.0095222

**Published:** 2014-04-16

**Authors:** Lígia S. Nobre, Ricardo Garcia-Serres, Smilja Todorovic, Peter Hildebrandt, Miguel Teixeira, Jean-Marc Latour, Lígia M. Saraiva

**Affiliations:** 1 Instituto de Tecnologia Química e Biológica António Xavier, Universidade Nova de Lisboa, Av. da República (EAN), Oeiras, Portugal; 2 Technische Universität Berlin, Institut für Chemie, FG Biophysikalische Chemie, Berlin, Germany; 3 DSV/iRTSV/CBM, UMR 5249 CEA-Université Grenoble I-CNRS/Equipe de Physicochimie des Métaux en Biologie, CEA-Grenoble, France; National Research Council of Italy (CNR), Italy

## Abstract

*Escherichia coli* RIC (Repair of Iron Centers) is a diiron protein previously reported to be involved in the repair of iron-sulfur proteins damaged by oxidative or nitrosative stresses, and proposed to act as an iron donor. This possible role of RIC was now examined specifically by evaluating its ability to donate iron ions to apo-iron-sulfur proteins, determining the iron binding constants and assessing the lability of its iron ions. We show, by UV-visible, EPR and resonance Raman spectroscopies that RIC may participate in the synthesis of an iron-sulfur cluster in the apo-forms of the spinach ferredoxin and IscU when in the presence of the sulfide donating system IscS and L-cysteine. Iron binding assays allowed determining the as-isolated and fully reduced RIC dissociation constants for the ferric and ferrous iron of 10^−27^ M and 10^−13^ M, respectively. Mössbauer studies revealed that the RIC iron ions are labile, namely when the center is in the mixed-valence redox form as compared with the (μ-oxo) diferric one. Altogether, these results suggest that RIC is capable of delivering iron for the formation of iron-sulfur clusters.

## Introduction

Iron-sulfur (Fe-S) clusters are among the most ancient and functionally versatile prosthetic groups in nature that underpin the action of multiple proteins involved in key metabolic pathways. In bacteria, two major systems assist the assembly of Fe-S clusters, namely the housekeeping ISC (Iron Sulfur Cluster) and the stress dedicated SUF (SUlFur assimilation) systems. To build and transfer the nascent Fe-S cluster to the target apo-protein both machineries require the action of pyridoxal-phosphate-dependent cysteine desulfurases (IscS or SufS), which catalyze the mobilization of a sulfur atom from cysteine, of scaffold proteins (IscU, SufU) in which the cluster is initially assembled, and of A-type carriers (IscA, SufA). A-type carriers IscA/SufA have also been reported to bind iron and proposed to act as iron donors for the assembly of Fe-S clusters in IscU/SufU. The exact molecular mechanisms for these processes are still under discussion, a major issue being the identity of the iron donor (reviewed in [1], [2]). In fact, besides IscA and SufA, frataxin has also been proposed to be an iron donor [3]. Apart from the ISC and SUF systems, *E. coli* encodes a Repair of Iron Centers (RIC) protein which was previously demonstrated to restore the activity of oxidatively and nitrosatively-damaged Fe-S enzymes, such as fumarase and aconitase [4]. The protein was initially called YtfE according to its gene name (*ytfE*), and was first remarked by its mRNA transcript being one of the most highly transcribed (∼55-fold increase) in anaerobically grown cells of *E. coli* treated with NO gas [5]. Highlighting its role in nitrosative and oxidative stress protection, the induction of the *ytfE* transcription was also observed in aerobically grown cells exposed to other NO donors [6], [7] and to hydrogen peroxide (our unpublished results).

Homologues of *E. coli* RIC have a widespread distribution in nature, including in Eukarya [8], [9] and their importance in the repair of damaged Fe-S centers was also shown for the human pathogens *Staphylococcus aureus and Neisseria gonorrhea* [8]. *In vivo* assays revealed that RIC contributes to the survival of *Haemophilus influenza* in activated macrophages [10].

The study of *E. coli* and *S. aureus* RICs proved that the proteins contain a diiron center of the histidine/carboxylate type [8], [11], [12], as it was initially proposed [11]. The electron paramagnetic resonance (EPR) spectrum of the partially reduced protein is characteristic of the S = ½ state of a mixed valence and antiferromagnetically coupled FeIII-FeII form of a binuclear iron center. The resonance Raman spectrum of the oxidized *E. coli* RIC displays a band at 490 cm^−1^, attributed to a symmetric Fe–O–Fe stretching mode of the μ-oxo-bridged diiron center, which on the basis of our EXAFS data was proposed to be further coordinated by one or two μ-carboxylate bridges from aspartate or glutamate residues plus histidines [12].

The damage that Fe-S centers suffer under stress depends on the type of cluster and on the stress inflicted (oxidative or nitrosative) but, in all cases, the rebuilding process requires re-insertion of iron [13], [14]. Since RIC was shown to be involved in repair of Fe-S clusters damaged by both stresses, we previously proposed that RIC may act as an iron donor [9]. In this work, the ability of RIC to donate iron for the assembly of an Fe-S center into the apo-form of spinach ferredoxin and of the *E. coli* scaffold IscU was analyzed by UV-visible, EPR and resonance Raman spectroscopies. The finding that RIC allows mounting of a Fe-S center led us to determine the iron dissociation constants and to study the lability of the iron ions of the diiron center using Mössbauer spectroscopy.

## Material and Methods

### Production of recombinant proteins

*E. coli* RIC was produced in *E. coli* BL21(DE3) Gold (Stratagene) transformed with pET-*ytfE* [11]. To this end, cells were grown aerobically in a 10-L fermenter, at 30°C, in M9 minimal medium with 20 mM glucose and 100 µM FeSO_4_. When cells reached an OD_600_∼0.3, protein expression was induced with 200 µM isopropyl-1-thio-β-D-galactopyranoside (IPTG), and the growth continued for 7 h. Cells expressing RIC were disrupted in a French Press, ultracentrifuged, and the soluble extract loaded into a Q-Sepharose High-Performance column equilibrated with 20 mM Tris-HCl pH 7.5. A linear salt gradient (0–1 M NaCl) in 20 mM Tris-HCl pH 7.5 at 2.5 mL/min was then applied and the fraction containing RIC eluted at ∼200 mM NaCl. This fraction was then introduced into a Superdex S-75 gel filtration column, previously equilibrated in 20 mM Tris-HCl plus 150 mM NaCl, pH 7.5 (buffer A). From this column a pure protein was obtained, as judged by gel electrophoresis.

*E. coli* IscS and apo-IscU proteins were produced fused to a His-tag at the N- and C-terminal, respectively, in *E. coli* M15:pREP4 cells harboring plasmids pQE30-(His)6-IscS and pQE60-IscU-(His)6, and purified as previously described [15].

The purity of all proteins was confirmed by SDS-PAGE, their concentration assayed by the bicinchoninic acid method [16] and the iron content determined by the TPTZ (2,4,6-tripyridyl-1,2,3-triazine) method [17]. The concentration of RIC protein was determined using the extinction coefficient ε_280nm_ = 24.26 mM^−1^ cm^−1^, which was obtained from an accurate protein quantification done by amino acid analysis performed at the PANATecs Protein Analytics Service (Germany).

All RIC fractions used in the present studies contained approximately 2 iron ions (1.8±0.3) per monomer.

### Monitoring the assembly of Fe-S clusters by UV-visible spectroscopy

Spinach ferredoxin (Sigma) was incubated with trichloroacetic acid (TCA, 10%) on ice for 30 min. Then, the protein was centrifuged, the pellet washed with TCA (1%), and desalted in a Micro Bio-Spin Chromatography column (BioRad) equilibrated in buffer A to yield apo-ferredoxin. The reconstitution of the [2Fe-2S]^2+/1+^ center into ferredoxin was achieved by anaerobic incubation of apo-ferredoxin (25 µM) with *E. coli* IscS (2.5 µM), L-cysteine (3 mM, Sigma), dithiothreitol (DTT, 10 mM, Sigma) and *E. coli* RIC (50 µM) in buffer A, in reactions that lasted for 15, 30 and 75 min. The reaction was initiated by the addition of L-cysteine and the amount of Fe-S cluster reconstituted in ferredoxin was estimated using the absorbance of the 415 nm band (ε = 9.4 mM^−1^cm^−1^) [18].

The formation of the Fe-S cluster in *E. coli* IscU was achieved by mixing in buffer A, the *E. coli* apo-IscU (50 µM) with IscS (4 µM), DTT (4 mM), and *E. coli* RIC (150 µM). The reaction was initiated by addition of L-cysteine (3 mM), and let proceed for 105 min under anaerobic conditions.

In both assays, control reactions were carried in the absence of the apo-recipient proteins (apo-ferredoxin and IscU). All the UV-visible spectra were recorded at room temperature in a Shimadzu UV-1700 spectrophotometer and the final spectra resulted from subtraction of the spectrum of a control sample mixture that contained all components and was acquired immediately after addition of cysteine (time zero).

### Electronic paramagnetic resonance analysis

The formation of the Fe-S center in ferredoxin was also analyzed by Electron paramagnetic resonance EPR. Apo-ferredoxin was prepared as described above. All the reconstitutions were made in the presence of 30 µM apo-ferredoxin, 4 mM DTT, 2 µM IscS, 2 mM L-cysteine and 300 µM *E. coli* RIC. The spectra of control reactions that contained all components except apo-ferredoxin or RIC were acquired. Two other control reactions in which RIC was replaced by ferrous iron (200 µM) or apo-RIC (300 µM) were also analyzed. In all the assays, the reaction was initiated by the addition of L-cysteine and let proceed for 2 h in an anaerobic chamber, after which, samples were transferred to EPR tubes and frozen in liquid nitrogen. After recording their spectra, samples were thawed (under anaerobic conditions) and reduced by the addition of a few drops of sodium dithionite solution (100 mM), frozen and analyzed again. EPR spectra were obtained on a Bruker EMX spectrometer equipped with an Oxford Instruments continuous flow helium cryostat and were recorded at 9.34 MHz microwave frequency, 2.0 mW microwave power, 1 mT modulation amplitude and at 17 K.

### Resonance raman studies

For the resonance Raman studies, two independent reaction mixtures containing IscU (50 µM), IscS (4 µM), DTT (4 mM), L-cysteine (3 mM), and RIC (150 µM) were incubated anaerobically, for 2.5 h. The samples were next concentrated in an ultrafiltration cell (Vivaspin 500, Vivascience Sartorius group) to a final concentration of ∼2 mM IscU, and introduced in a cryostat (Linkam) mounted on a microscope stage. Spectra were recorded at −190°C from droplets of frozen samples in backscattering geometry by using a confocal Raman microscope (JobinYvon, XY), equipped with 1200 l/mm grating and a liquid-nitrogen-cooled back-illuminated CCD detector. The 457-nm line from an Argon ion laser (Coherent Innova 70) was used for excitation, with the laser power at the sample set to 13 mW and accumulation time of 40 seconds. A control sample containing all components, except RIC, was also analyzed.

### Mössbauer studies

The recombinant *E. coli* ^57^Fe-RIC was produced and purified as described above, but using as iron source ^57^FeSO_4_ (100 µM) in the growth medium. A second sample was also studied by Mössbauer spectroscopy, which was prepared by incubating overnight under anaerobic conditions and at room temperature, ^56^Fe-RIC with six molar equivalents of ^57^FeSO_4_ in buffer A containing 2 mM DTT, after which the mixture was passed through a desalting column. The two samples were concentrated up to ∼1 mM, loaded into Mössbauer cuvettes and frozen with liquid nitrogen. Mössbauer spectra were recorded at 4.2 K, either with a low-field Mössbauer spectrometer equipped with a Janis SVT-400 cryostat, or with a strong-field Mössbauer spectrometer equipped with an Oxford Instruments Spectromag 4000 cryostat containing an 8T split-pair superconducting magnet. Both spectrometers were operated in a constant acceleration mode in transmission geometry. The isomer shifts are referenced against that of a metallic iron foil at room-temperature. Analysis of the data was performed with the program WMOSS (WEB Research, Edina, MN, USA).

### Iron association assays

The ferric and ferrous iron ions dissociation constants of *E. coli* RIC were determined for the as-isolated and fully reduced form of the protein. Fully reduced RIC was obtained by addition, under anaerobic conditions, of a threefold molar excess of sodium dithionite (Sigma) in 50 mM Tris- HCl pH 8 buffer. Due to the difficulty of fully oxidizing RIC, the iron dissociation constant of the fully oxidized form was not determined.

To evaluate the dissociation constant of iron from RIC in water (Kd_RIC_), defined by Kd_RIC_  =  [Fe][RIC]/[RIC-Fe], a competitive assay was done using two iron chelators and recorded by UV-visible spectroscopy. The as-isolated RIC (25 µM) was incubated, in buffer A, overnight at room temperature under aerobic conditions, with several concentrations of desferrioxamine (5–1000 µM) (Sigma) to remove ferric iron. The as-isolated and reduced RIC (25 µM) were anaerobically incubated, in buffer A, overnight and at room temperature, with several concentrations of 2,2′-dipyridyl (5–1000 µM) (Sigma) to remove ferrous iron. For the two chelators, each concentration was performed independently and in triplicate. The overnight incubation proved essential to achieve thermodynamic equilibrium thus allowing a precise determination of the dissociation equilibrium constants.

The Kd_RIC-chelator_, defined by Kd_RIC-chelator_  =  [RIC-Fe][Chelator]/[Chelator-Fe][RIC], is the dissociation constant of iron from RIC in the presence of the competing chelator. The constant was determined using the equation for a saturation binding experiment with one specific binding site: Y = B_max_*X/(Kd_RIC-chelator_ +X), in which X is the concentration of the chelator, Y the percentage of iron atoms released and B_max_ the maximum percentage of iron atoms released (GraphPad Prism version 5 for Windows, GraphPad Software, San Diego California USA, www.graphpad.com). The percentage of iron atoms released was determined by measuring the absorbance of the iron-chelator complex and assuming that the initial concentration of the iron-bound RIC is 100%. The following extinction coefficients were used: ε_420nm_ = 2.9×10^3^ M^−1^cm^−1^ and ε_522nm_ = 8.7×10^3^ M^−1^cm^−1^ for desferrioxamine-Fe(III) and 2,2′-dipyridyl-Fe(II), respectively [19], [20]. This model could be used as the data did not indicate, within the experimental errors, a biphasic behavior attributable to the two iron ions of the cluster. Hence, at this stage and as a first approximation, it was assumed that both sites have identical binding constants and that they behave independently of each other.

The actual dissociation constant of iron from RIC, Kd_RIC_, was then calculated using the equation Kd_RIC_  =  1/(Kd_RIC-chelator_ x Ka_chelator_), in which Ka_chelator_  =  [Chelator-Fe]/[Chelator][Fe] and correspond to the affinity constants of desferrioxamine-FeIII (∼10^31^ M^−1^) or 2,2′-dipyridyl-FeII (10^17^ M^−1^), respectively [21], [22].

## Results

### Assembly of Fe-S centers in the presence of E. coli RIC

To investigate the ability of RIC to act as an iron donor, RIC was mixed with apo forms of the recipient proteins, spinach ferredoxin and *E. coli* IscU, in the presence of the sulfide donor system IscS and L-cysteine, under anaerobic conditions. The reconstitution of the Fe-S cluster was monitored by UV- visible, EPR and resonance Raman spectroscopies.

### Reconstitution of the Fe-S center of ferredoxin

**Figure 1:**
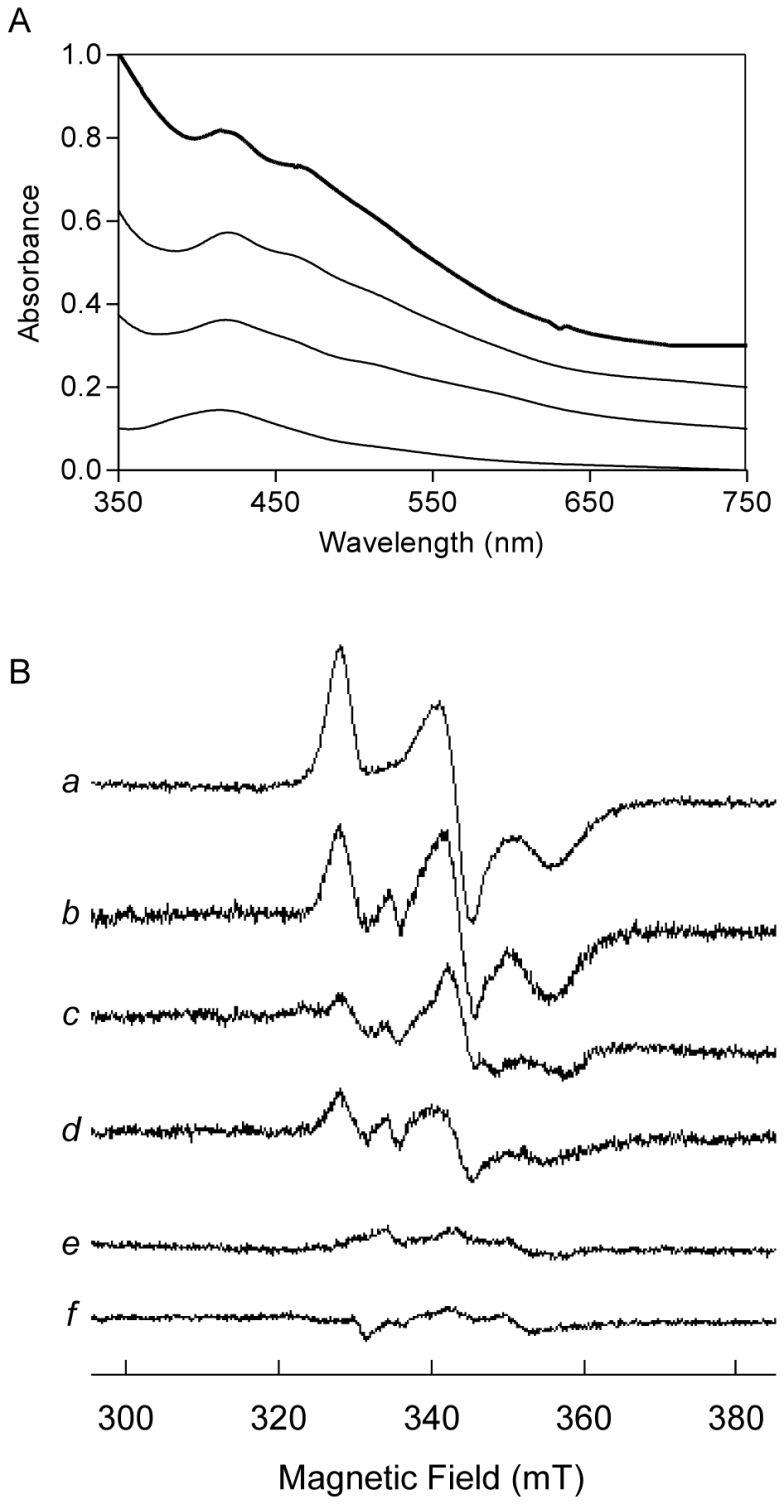
Formation of Fe-S clusters in apo-ferredoxin mediated by RIC. **A**. UV-Visible spectra of ferredoxin upon incubation, under anaerobic conditions, of apo-ferredoxin (25 µM) with RIC (50 µM), IscS (2.5 µM), and L-cysteine (3 mM) for 15, 30 and 75 min (from bottom to top). Spectra are offset for clarity and the top bold-line spectrum corresponds to the commercial spinach holo-ferredoxin. **B**. EPR spectra of reduced holo-ferredoxin (30 µM) (trace *a*) and of reconstitution reactions of apo-ferredoxin (30 µM) achieved in 20 mM Tris-HCl plus 150 mM NaCl, pH 7.5 buffer containing 4 mM DTT, 2 µM IscS and 2 mM L-cysteine using as iron source 300 and 100 µM *E. coli* RIC (traces *b* and *c*, respectively) and 200 µM ferrous iron (trace *d*). The spectra of the reaction mixture prepared as in *b* but lacking the apo-ferredoxin (trace *e*) or done with apo-RIC (trace *f*) are also presented. Spectra were recorded at 9.34 MHz microwave frequency, 2.0 mW microwave power, 1 mT modulation amplitude and temperature 17 K.

The formation of the Fe-S center in ferredoxin assisted by RIC was first monitored by UV-visible spectroscopy. Anaerobic incubation of the apo-ferredoxin (25 µM) with RIC (50 µM) was done in the presence of DTT, IscS and L-cysteine. The final reaction mixture presented a brownish color and a visible spectrum with major bands at 415 and 460 nm (Figure 1A); starting with the apo-ferredoxin, the absorbance at those wavelengths increased with time and reached a maximum after 75 min. These bands are characteristic of a [2Fe-2S]^2+/1+^ center typically present in the oxidized form of the spinach ferredoxin (Figure 1A, bold line). This spectrum shows that under the conditions used the binuclear center was oxidized, presumably a result of its quite low reduction potential (−400 mV, [23]), and in agreement with the EPR data described below. Considering the absorption of the 415 nm band of the spectrum recorded after 75 min, we determined that approximately one (ca 70%) Fe-S center was reconstituted in the spinach ferredoxin.

The formation of the Fe-S center of ferredoxin in a reaction containing DTT, IscS, L-cysteine and *E. coli* RIC was also analyzed by EPR spectroscopy. The spectrum of oxidized ferredoxin is EPR silent (data not shown), indicating, in concordance with the optical data, that the Fe-S centers are oxidized; therefore, all the samples were reduced by the addition of dithionite and analyzed before and after reduction. The rhombic species observed for reduced reconstituted ferredoxin, obtained in the presence of *E. coli* RIC or ferrous iron, with *g* values of 1.89, 1.92 e 2.04, is identical to that of the reduced native protein (Figure 1B). No reconstitution of the Fe-S center was observed in control reactions containing all components except apo-ferredoxin (spectrum *e*), or done in the absence of *E. coli* RIC (data not shown as resonances were not observed). Identically, the Fe-S center in ferredoxin was not formed when the reaction was performed with apo-RIC (Figure 1B spectrum *f*). Using the EPR spectrum of native ferredoxin as a reference, it could be concluded that the reconstitution of the Fe-S center in the presence of RIC amounted up to ca. 75%.

### Assembly of the Fe-S center in IscU

**Figure 2:**
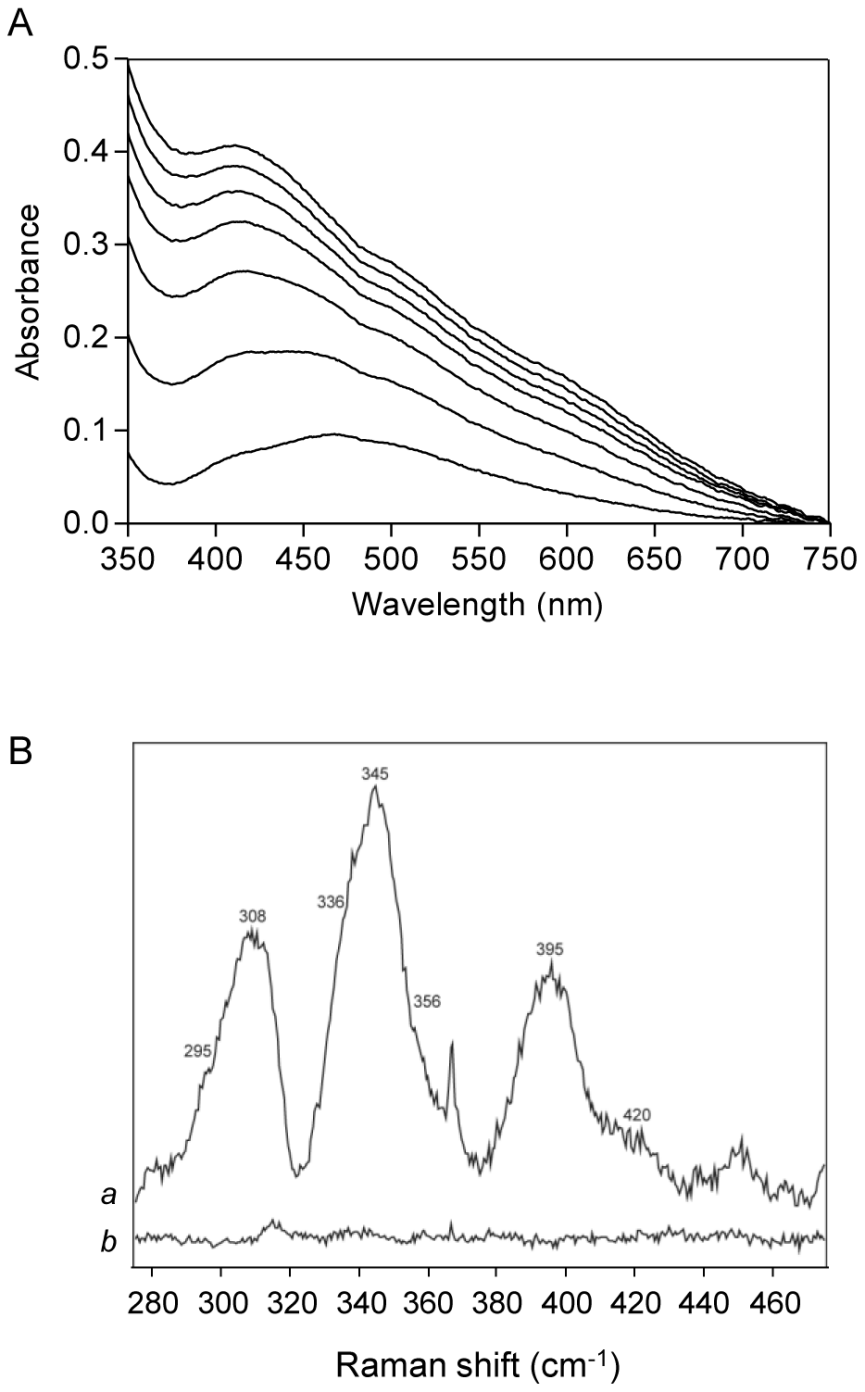
UV-visible and Resonance Raman spectra of the Fe-S cluster formed in *E. coli* IscU via RIC. **A**. Visible spectra of IscU (50 µM) depicting the Fe-S cluster formed upon anaerobic incubation with RIC (150 µM), IscS (4 µM) and L-cysteine (3 mM). The spectra were collected every 15 min for a period of 105 min (bottom to top). **B**. Resonance Raman spectra of a reaction mixture containing IscU (50 µM), L-cysteine (3 mM), IscS (4 µM), DTT (4 mM) and RIC (150 µM) (*a*), and of a reaction mixture with all components, except RIC (*b*). Spectra were acquired at −190°C with 457 nm excitation, a laser power of 13 mW and accumulation time of 40 s.

Similar experiments were performed using as recipient *E. coli* apo-IscU, a scaffold protein that is endowed with the ability to assemble [2Fe-2S]^2+/1+^ and [4Fe-4S]^2+/1+^ clusters [24]. To achieve this, apo-IscU (50 µM) was incubated anaerobically with RIC (150 µM) in buffer A containing L-cysteine, IscS and DTT, and the reaction was followed overtime by visible spectroscopy (Figure 2A). After 45 min, the spectrum exhibited two bands centered at 456 and 410 nm characteristic of the formation of a [2Fe-2S]^2+/1+^ center [24], [25]. For longer times, the spectrum broadened and after 75 min the 410 nm band became dominant (Figure 2A), with spectral features more similar to those of a [4Fe-4S]^2+/1+^ cluster containing IscU [26]. Based on the 456 nm band, for transient Fe-S clusters in IscU [25], the UV-visible spectrum of the reaction mixture obtained at 45 min, suggests that a [2Fe-2S]^2+/1+^ cluster was assembled in IscU. At longer incubation times, namely after 75 min, and as previously observed [24] there is the formation of [4Fe-4S]^ 2+/1+^ centers, as evidenced by the band around 400 nm.

The presence of the Fe-S cluster in *E. coli* IscU promoted by RIC was also analyzed by resonance Raman spectroscopy. To this end, apo-IscU (50 µM) was incubated with RIC (150 µM) in a reaction mixture that also contained L-cysteine and IscS. A control reaction mixture with no RIC was also prepared, and the spectrum of each reaction mixture was obtained using an excitation laser line at 457 nm. Under these experimental conditions, the resonance Raman bands originating from RIC do not contribute to this frequency region [12]. The spectrum of the reaction mixture containing RIC shows several bands around 340 cm^−1^ (Figure 2B, spectrum a) due to oxidized Fe-S clusters, which are the only ones detected by resonance Raman. These bands are absent from the spectrum of the control reaction done in the absence of RIC (Figure 2B, spectrum b). Vibrational modes at 295, 345, 395 and 420 cm^−1^ fall into the range of frequencies characteristic of oxidized [2Fe-2S]^2+/1+^ clusters [12], [24], [27]-[29]; the bands at 295 cm^−1^ and 345 cm^−1^ correspond to the terminal modes B_3u_ and A_g_, respectively, and the 396 cm^−1^ and 420 cm^−1^ bands to A_g_ and B_2u_ bridging modes of a newly formed [2Fe-2S]^2+/1+^ oxidized cluster in *E. coli* IscU [30]. The shoulders at 336 and 356 cm^−1^ are indicative of bridging and terminal modes of oxidized [4Fe-4S]^2+/1+^ clusters, and these bands were also previously observed in the spectrum of *E. coli* IscU after a reconstitution reaction promoted by frataxin (CyaY) [29]. The modes assigned to the [2Fe-2S]^2+/1+^ center show slight downshifts in comparison with those reported for *E. coli* IscU [29], but are consistent with the energies of vibrational modes observed in other [2Fe-2S]^2+/1+^ clusters [12], [24], [27], [28], [30]. The origin of the intense band at 308 cm^−1^ is not clear at this point; nevertheless, it might be due to contribution from a non-resolved ice lattice mode [31].

Note that during the reconstitution of the Fe-S center in ferredoxin and in IscU the formation of iron-sulfides was not observed in the reaction mixtures as indicated by the absence of bands at ∼500 and 600 nm typically present in UV-visible spectra resulting from mixing sulfide and ferrous iron [32]. Furthermore, in the control reactions performed without apo-recipient proteins, no assembly of a Fe-S center was observed (Figure S1 and Figure 1B, spectrum *e*).

### Release of iron from *E. coli* RIC

**Figure 3:**
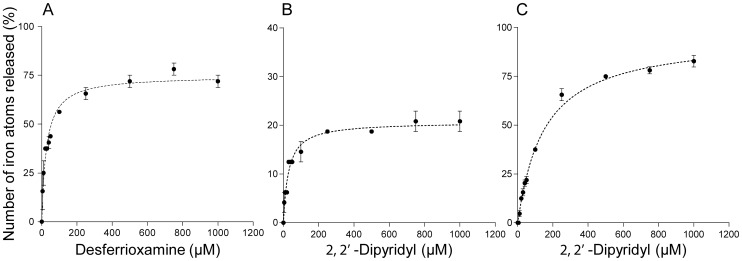
Demetalation of RIC iron with iron chelators. Independent samples of the as-isolated *E. coli* RIC (25 µM) (A,B) and of fully reduced RIC (C) were mixed with increasing concentrations of desferrioxamine (A) and 2,2′ dipyridyl (B and C) and incubated overnight (∼18 h). The absorbance of the bands at 420 and 522 nm were used to calculate the percentage of ferric and ferrous iron atoms released from RIC as described in Material and Methods. Results are mean of three independent assays with error bars representing standard errors.

The above data indicates that RIC donates iron for the reconstitution of Fe-S centers. To further evaluate this process, we determined the iron binding dissociation constants of RIC in the as-isolated and fully reduced forms. Desferrioxamine and 2,2′-dipyridyl were used as FeIII and FeII chelators, respectively, and the intensities of the 420 nm absorption band for the desferrioxamine-FeIII complex and of the 522 nm band for the dipyridyl-FeII complex were utilized to calculate the amount of iron released. Furthermore, several concentrations of chelator were tested in independent reaction mixtures that were incubated under aerobic and anaerobic conditions for desferrioxamine and 2,2′- dipyridyl, respectively (Figure 3).

Incubation of the as-isolated RIC with each chelator released approximately 74±3% of FeIII and 21±2% of FeII with similar dissociation constants (Kd_RIC-chelator_) of ∼30×10^−6^ M towards the chelators (Figure 3A–B, Table 1). Anaerobic incubation of the fully reduced form of RIC with 2,2′-dipyridyl led to the release of 96±4% of FeII with a dissociation constant of 151±11×10^−6^ M in relation to the chelator (Figure 3C and Table 1). Furthermore, the data indicate that the two iron sites have similar dissociation constants.

**Table 1:** Iron dissociation constants of *E. coli* RIC.

RIC	Kd_RIC-chelator_ (M)*		Kd_RIC_ (M)		Percentage of iron released	
	FeII	FeIII	FeII	FeIII	FeII	FeIII
As-isolated	30±13×10^−6^	29±5×10^−6^	4±1×10^−13^	4±1×10^−27^	21±2	74±3
Reduced	151±11×10^−6^	-	7±1×10^−14^	-	96±4	-

*Desferrioxamine and 2,2′-dipyridyl were used as chelators of FeIII and FeII, respectively.

The constants associated with the binding of iron ions to RIC (Kd_RIC_) were then determined using the values above indicated (Kd_RIC-chelator_) and the equations described in Material and Methods. The data showed that in the as-isolated RIC, the FeIII is more tightly bound (Kd_RIC_ = 4±1×10^−27^ M) than FeII (Kd_RIC_  = 4±1×10^−13^ M). Moreover, the strength of the FeII binding does not vary significantly when the protein is fully reduced, in which case the Kd_RIC_ is 0.7±1×10^−13^ M (Table 1).

### Mössbauer study of the diiron center of RIC

*E. coli* RIC contains a diiron center and the as-isolated protein is a mixture of at least two forms, the oxidized and the mixed valence one [11]. Mössbauer spectroscopy was used to characterize the various redox forms of the diiron center present in the as-isolated protein and to rationalize the iron dissociation experiments. Furthermore, we have investigated the lability of the iron ions by incubation of ferrous ^57^Fe with a sample of holo-RIC containing natural-abundance iron, and therefore virtually silent in Mössbauer spectroscopy.

**Figure 4:**
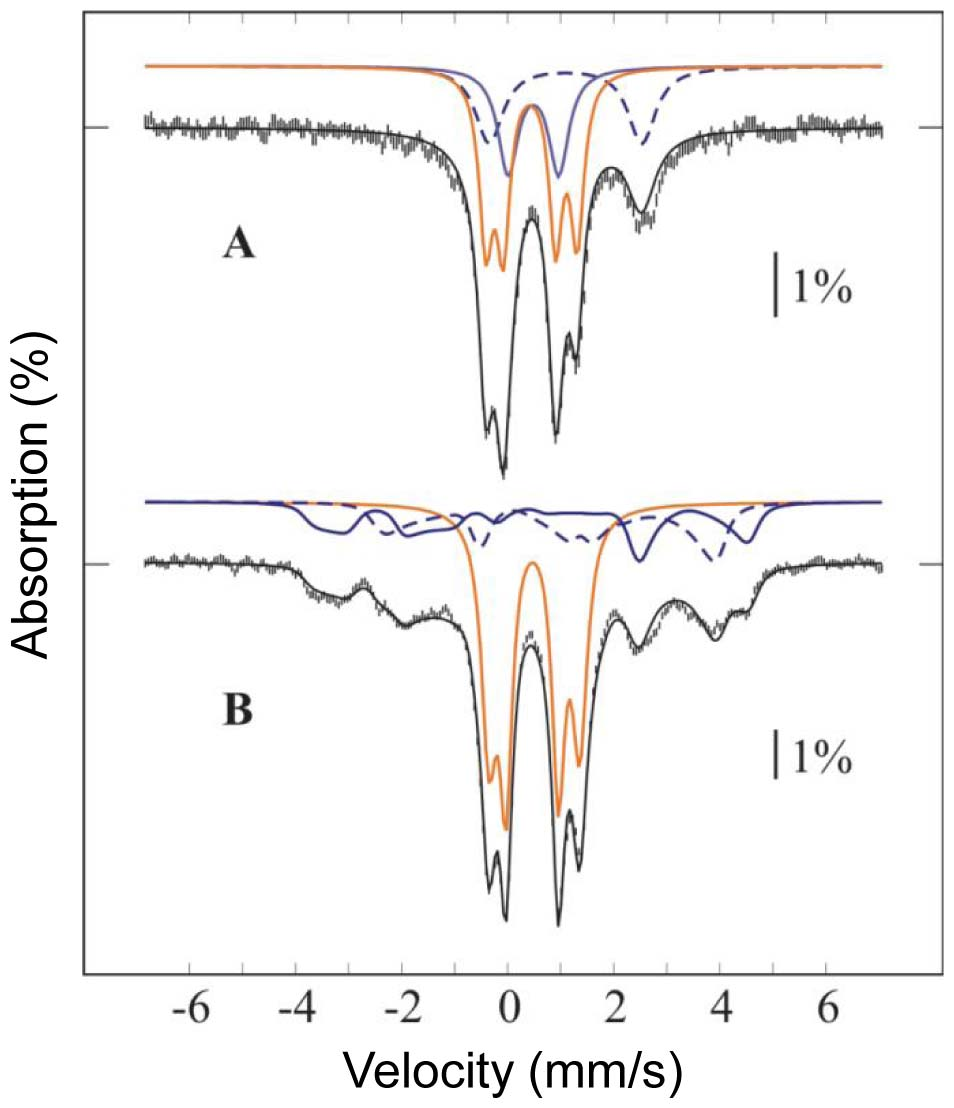
Mössbauer spectrum of the as-isolated *E. coli* RIC. The spectrum was acquired at 4.2 K in a 600 G magnetic field applied perpendicular to the γ-rays (A) and at 154 K with no applied field (B). Vertical bars represent the experimental spectra, while the black solid line overlaid with the data represent the theoretical spectra. Contributions from mixed-valence (blue), and from diferric (orange) RIC are plotted above the experimental data. In the mixed-valence contribution, the trace originating from the FeIII is plotted as a blue solid line, whereas the trace from the FeII is plotted as a blue dotted line. The parameters used in the simulation are listed in Table 2, and the abundances of different iron sites in Table 3. The same abundances were used in the simulations of both spectra. For the mixed-valence form, the isomer shifts of the two iron sites obtained from fitting the 154 K spectrum were used into the spin-Hamiltonian simulation of the 4.2 K spectrum, after correction for second order Doppler shift (SODS). Quadrupole splittings were found to be substantially different and were fit separately. In contrast, the diferric component displays no temperature dependence of either isomer shifts (other than SODS) or quadrupole splittings, and displays slightly smaller line widths at higher temperature, indicating an isolated S = 0 ground state.

The 4.2 K low-field Mössbauer spectrum of as-isolated ^57^Fe-RIC (Figure 4B) displays two main quadrupole doublets centered at 0.5 mm/s, representing altogether 56% of total iron, and a magnetically-split component accounting for the remaining iron. The two main absorptions could be simulated with two equal-intensity quadrupole doublets with isomer shifts typical for N/O-bound octahedral high-spin FeIII (Table 2). The observation of doublets in these recording conditions suggests that the two FeIII ions are antiferromagnetically coupled. Moreover, spectra acquired at high-applied magnetic field (data not shown) confirmed the diamagnetic ground state (S = 0 total spin) of the diiron site associated with these two doublets. The high quadrupole splittings of the two doublets (0.99 and 1.71 mm/s) are in line with those observed for μ-oxo bridged diferric proteins [33]. The occurrence of a hydroxo-bridged diferric center can be eliminated because (i) they generally have quadrupole splittings significantly smaller than 1 mm/s [34] and (ii) no broadening of the Mössbauer lines was observed at higher temperatures (Figure 4A), as it generally occurs with the weaker coupling from hydroxo bridges [35]–[37]. In agreement with our previous Raman and EXAFS data [12], Mössbauer spectroscopy indicates unambiguously the presence of a μ-oxo-bridged diiron center in *E. coli* RIC.

**Table 2:** Spin-Hamiltonian and quadrupole doublet parameters used in the 4.2 K Mössbauer simulations.

Type of center	Spin	δ (mm/s)	ΔE_Q_ (mm/s)	η	A (T)	Γ (mm/s)
Diferric						
A	0	0.46	0.99			0.35
B		0.50	1.71			0.34
Mixed-valence						
FeIII	1/2	0.52	−1.45	0.26	−44, −44, −44	0.35
FeII		1.15	+2.96	0.09	32, 28, 14	0.35
Diferrous		1.14	3.50			0.40

Parameters: δ –isomeric shift, η – asymmetry parameter, ΔE_Q_ –quadrupole splitting; Γ – line width; A- hyperfine constant.

The diferric and mixed-valence parameters were determined from as-isolated RIC, while the diferrous parameters were determined from reduced RIC (data not shown).

The magnetically-split component is clearly associated with a half-integer spin system. A spectrum measured at 154 K (Figure 4A) was used to determine the isomer shifts of both iron sites, taking advantage of the signal collapsing into quadrupole doublets due to fast electronic relaxation. After correction of the isomer shifts for second order Doppler effect, this component was simulated with an effective S = 1/2 spin Hamiltonian, as the sum of two equal-intensity sub-spectra, corresponding to an antiferromagnetically coupled mixed-valence FeII-FeIII pair. Preliminary fit of several spectra measured at 4.2 K in different applied magnetic fields (data not shown) afforded the spin-Hamiltonian parameters listed in Table 2, which are typical for the mixed valence state of diiron proteins [35]–[38]. A good simulation of the overall spectrum was obtained by considering that 56% of total Fe is in the (μ-oxo)diferric form and 44% in the mixed-valence FeII-FeIII state. These data indicate that the as-isolated protein has approximately 78% of iron in the ferric state (both in the oxidized and in the mixed valence states), and 22% in the ferrous form (in the mixed valence state) (Table 3). Noteworthy, all *E. coli* RIC samples used in the EPR [11] and Mössbauer studies did not contain iron mononuclear centers (Table 3 and Figure 4).

**Table 3:** Percentages of each form of iron relative to the total amount of ^57^Fe.

Diiron Center	As-isolated RIC	[^56^Fe RIC + ^57^Fe]
Diferric		
A	28	12
B	28	12
Mixed valence		
FeIII	22	51
FeII	22	15
Ferrous	-	10

**Figure 5:**
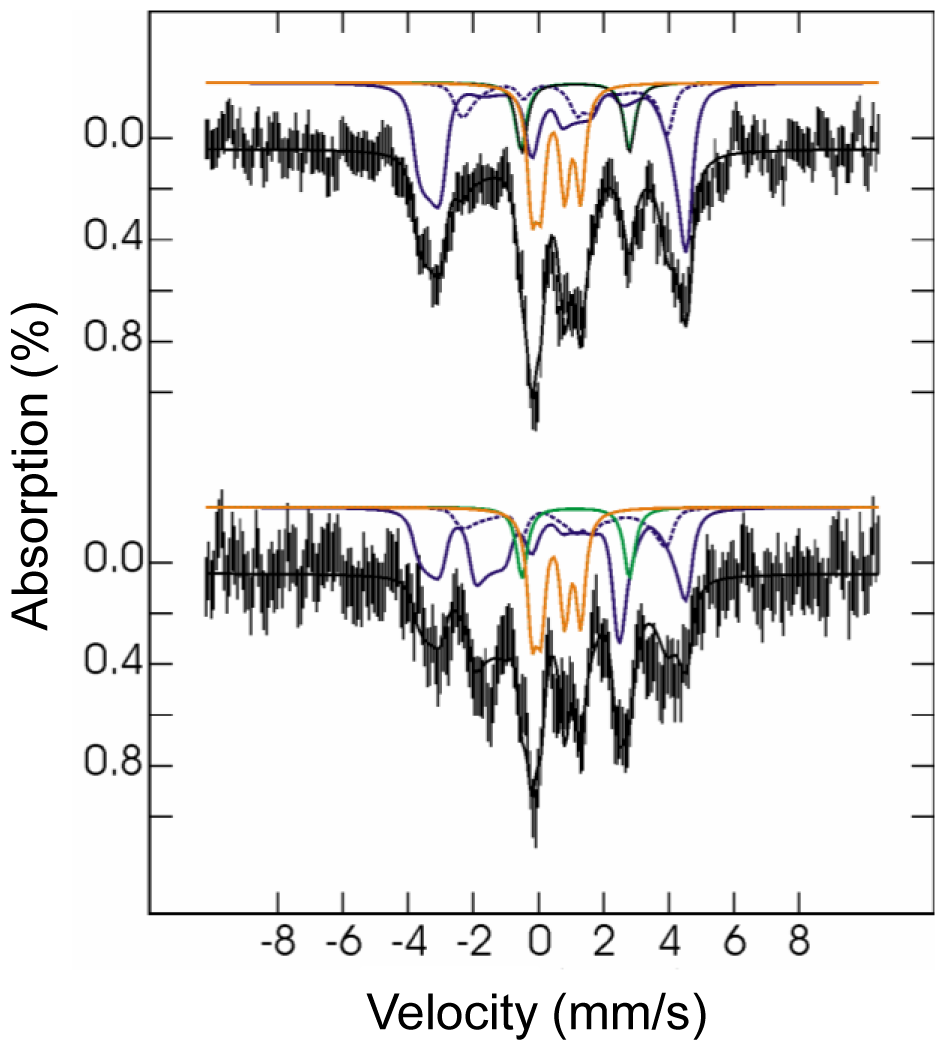
Mössbauer spectra of *E. coli* ^56^Fe-diiron RIC after incubation with ^57^Fe. Spectra were measured at 4.2 K in a 600 G magnetic field applied parallel (top) or perpendicular (bottom) to the γ-rays. Vertical bars represent the experimental spectra while black solid lines represent the theoretical spectra. Contributions from mixed-valence (blue), diferric (orange) and ferrous species (green) are plotted above the experimental data. Contributions in percentage of total simulated intensity are 10% of ferrous, 24% of diferric, 51% of Fe^III^ in mixed-valence centers (solid blue lines), and 15% of Fe^II^ in mixed-valence centers (dotted blue lines).

The sample resulting from the incubation of the ^56^Fe-RIC with ferrous ^57^Fe exhibited the Mössbauer spectrum depicted in Figure 5. Since ^56^Fe is Mössbauer-silent and the as-isolated RIC had an almost full occupancy of the diiron centers, the observation of the diiron center's spectral features showed that the added ^57^Fe has exchanged with labile iron sites, rendering them visible in the Mössbauer spectrum with parameters characteristic of RIC sites. It should be noted that the baseline of the spectrum is not completely flat, most probably due to the presence of a small amount of ferric aggregates, accounting at most 30% of total iron.

The spectrum was simulated with the parameters cited above but the ratios of the two Fe in the (μ-oxo)diferric and mixed valence centers were allowed to vary. Moreover the presence of an additional ferrous component was required to simulate the spectra. The fitting of the spectra yielded the following Fe contributions (Figure 5, Table 3): (μ-oxo)diferric centers 24% of FeIII, mixed valence centers 51% of FeIII and 15% of FeII and ferrous ions 10%. From these data it is not possible to discriminate adventitiously bound Fe^2+^ from ferrous RIC centers in the ^56^Fe-RIC incubated with ferrous ^57^Fe. The data show that 75% of ^57^FeIII and 15–25% of ^57^FeII were incorporated in the previously all ^56^Fe RIC diiron centers. This experiment clearly demonstrates the lability of the Fe ions of the RIC dinuclear center. It also shows that the mixed-valence center is more labile than the (μ-oxo)diferric center.

## Discussion

*E. coli* RIC harbors a diiron center that in the as-isolated protein occurs in a ferric and mixed valence states, as here shown by Mossbauer spectroscopy, due to its high reduction potential [4], [12]. This work reveals that RIC is able to donate iron ions to apo Fe-S proteins, an essential step for the Fe-S clusters repair process when the extension of the damage leads to the complete loss of the center. In fact, we showed that RIC allows the formation of a [2Fe-2S]^2+/1+^ cluster in spinach ferredoxin, while for IscU the initial formation of a [2Fe-2S]^2+/1+^ center is observed followed by the appearance of a [4Fe-4S]^2+/1+^ cluster after longer incubation times, similarly to what was previously reported when using this scaffold protein [24].

The analysis of the iron binding properties of the RIC diiron center, done in experiments that used metal-chelators, allowed estimating that the as-isolated *E. coli* RIC contains ∼75% of FeIII and ∼20% for FeII. Moreover, ∼94% of FeII was chelated by dipyridyl from the fully reduced form of RIC. These results agree with the iron composition determined by Mössbauer spectroscopy, which showed that 44% of the total iron is present in the mixed-valence state [FeIII-FeII] and 56% in the oxodiferric form [FeIII-FeIII], yielding ca. 78% and 22% of ferric and ferrous iron, respectively. In the as-isolated *E. coli* RIC, the FeII is less tightly bound (Kd_RIC_ ∼10^−13^ M) than FeIII (Kd_RIC_ ∼10^−27^ M). However, considering that the intracellular concentration of loosely bound iron is estimated as being ∼10 µM [39], the *in vivo* dissociation of the iron from RIC is therefore prevented and suggests that the release of iron should be triggered.

The assembly of Fe-S clusters requires a source of sulfur and iron and while cysteine desulfurases, represented in *E. coli* by IscS and SufS, are so far recognized as the only enzymes capable of providing sulfur, the iron source seems to be more versatile. Heretofore, *E. coli* CyaY, IscA, SufA, and *S. enterica* YggX have been proposed to provide iron for the *in vitro* maturation of Fe-S clusters. The available studies on the dissociation constants of these putative iron donors report a strong ferric iron affinity in *E. coli* IscA and CyaY (K_dIscA_ ∼10^−18^ M and K_dCyaY_ <10^−17^ M) and a weak ferrous iron binding in *S. enterica* YggX and *E. coli* IscA/SufA (K_d_ ∼10^−5^–10^−6^ M) [3], [40]–[42]. *E. coli* RIC exhibits a FeIII dissociation constant of ∼10^−27^ M showing that the ferric iron is tightly bound, as it occurs in IscA and CyaY. However, the RIC dissociation constant for ferrous iron (∼10^−13^ M) is lower than those of other potential ferrous iron donors, namely YggX, IscA and SufA. Based on this data, we propose that RIC acts as a ferrous iron donor. Moreover, our Mössbauer studies revealed that the iron in *E. coli* RIC is labile as addition of external ^57^Fe to an intact ^56^Fe-diiron containing protein results into incorporation of ^57^Fe into the diiron cluster.

Altogether, the data now obtained suggest that *E. coli* RIC may also act as an iron donor under non-stress conditions, which is consistent with the previous observation that even non-stressed *E. coli ytfE* mutant cells have lower aconitase and fumarase activity [11]. We speculate that under stress conditions, which induce partial or complete destruction of the clusters from the hydratases, RIC may interact with IscS/SufS to provide both the iron and the sulfur required for the reassembling process.

## Supporting Information

Figure S1 **UV-visible spectra of the control reaction for IscU reconstitution along time.** IscS (4 µM), L-cysteine (3 mM) and RIC (150 µM) were mixed, anaerobically, in 20 mM Tris-HCl, 150 mM NaCl, pH 7.5 buffer with 4 mM DTT (same reaction as in Fig. 2B, but without IscU). The spectra were collected every 15 min for a period of 105 min (bottom to top).
(DOCX)
