# Supplementary material for: Escherichia coli RIC Is Able to Donate Iron to Iron-Sulfur Clusters
Source: PLoS One. 2014 Apr 16;9(4):e95222. doi: 10.1371/journal.pone.0095222 (PMC3989283; doi:10.1371/journal.pone.0095222)
Supplement: Figure S1 — UV-visible spectra of the control reaction for IscU reconstitution along time. IscS (4 µM), L-cysteine (3 mM) and RIC (150 µM) were mixed, anaerobically, in 20 mM Tris-HCl, 150 mM NaCl, pH 7.5 buffer with 4 mM DTT (same reaction as in Fig. 2B, but without IscU). The spectra were collected every 15 min for a period of 105 min (bottom to top). (DOCX) [file pone.0095222.s001.docx]

**Figure S1**


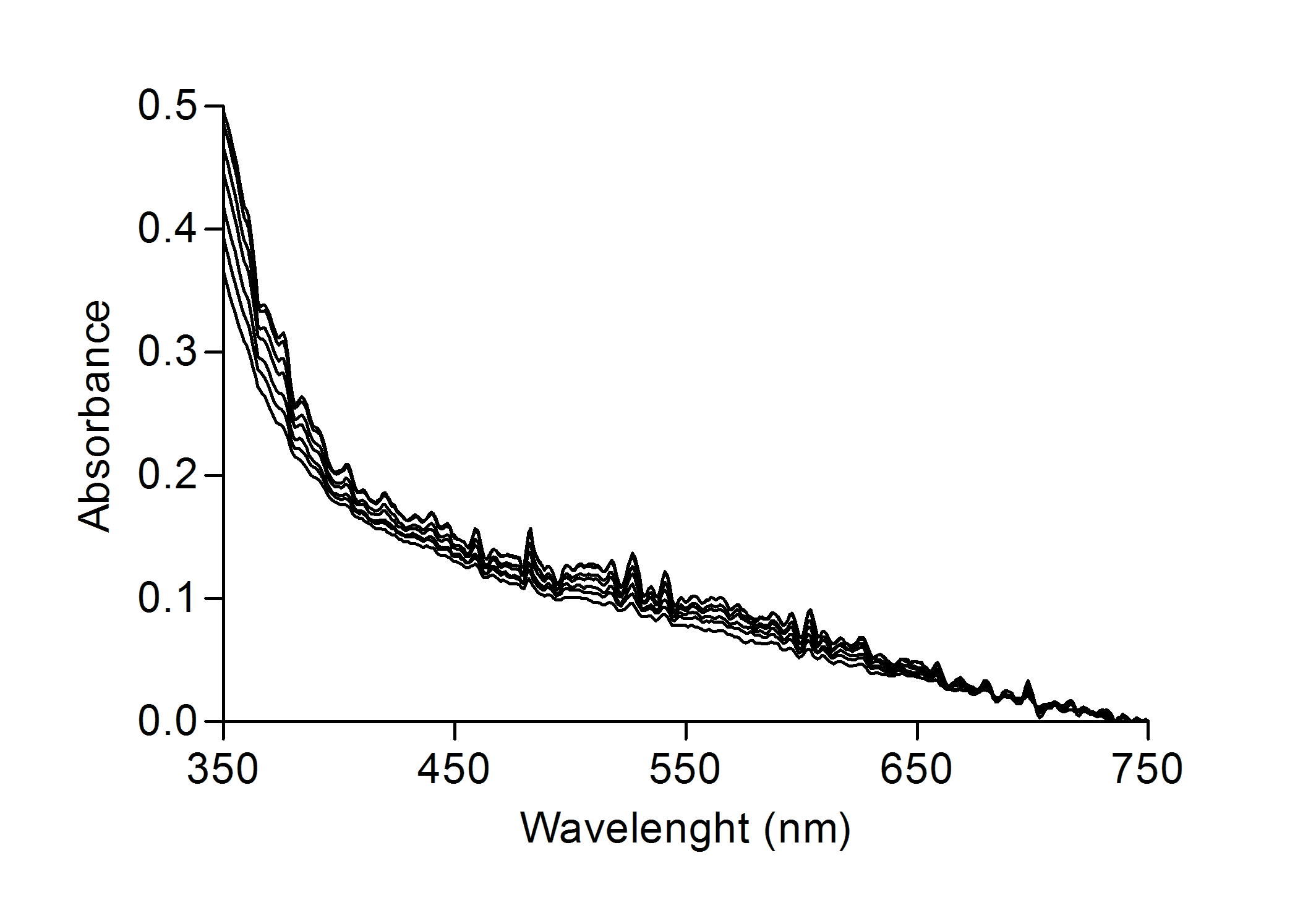


**Figure S1. UV-visible spectra of the control reaction for IscU reconstitution along time.**

IscS (4 µM), L-cysteine (3 mM) and RIC (150 µM) were mixed, anaerobically, in 20 mM Tris-HCl, 150 mM NaCl, pH 7.5 buffer with 4 mM DTT (same reaction as in Fig. 2B, but without IscU). The spectra were collected every 15 min for a period of 105 min (bottom to top).
